# From molecular advances to limited clinical impact: The persistent gap in chromoblastomycosis

**DOI:** 10.1371/journal.pntd.0014801

**Published:** 2026-10-13

**Authors:** Daniel Wagner C. L. Santos, Dallas J. Smith, Vania Aparecida Vicente, Bruno Paulo Rodrigues Lustosa, Conceição de Maria Pedrozo e Silva de Azevedo

**Affiliations:** 1 Division of Infectious Diseases of Hospital Universitário Presidente Dutra da Universidade Federal do Maranhão (UFMA), Ebserh, São Luís, Maranhão, Brazil; 2 Instituto D’Or de Pesquisa e Ensino, IDOR, São Paulo, Brazil; 3 Mycotic Diseases Branch, Centers for Disease Control and Prevention, Atlanta, Georgia, United States of America; 4 Microbiology, Parasitology, and Pathology Post Graduation Program, Department of Pathology, Federal University of Paraná, Curitiba, Brazil; 5 Department of Medicine, Federal University of Maranhão, São Luís, Maranhão, Brazil; 6 Post-graduation Program of Health Science, Federal University of Maranhão, São Luís, Maranhão, Brazil; Institute of Continuing Medical Education of Ioannina, GREECE

## Abstract

**Background:**

Chromoblastomycosis (CBM) is a chronic implantation mycosis of considerable morbidity, first described more than a century ago, predominantly affecting rural populations in tropical and subtropical regions. Although historically associated with impoverished settings, an increasing number of cases have been reported in both endemic and non-endemic areas, including high-income countries, reflecting an underrecognized epidemiological landscape. Despite its burden, key metrics such as disability-adjusted life years (DALYs) remain undefined, as highlighted by recent global health initiatives. While advances in diagnosing and treating patients with CBM have been made, new technologies have not been incorporated into broader clinical and public health interventions.

**Methodology/principal findings:**

Herein, we synthesize recent advances in CBM, focusing on the expanding biodiversity of etiological agents driven by molecular taxonomy, the growing role of molecular diagnostics, and emerging therapeutic strategies. Novel genera and species have been identified, particularly among immunocompromised hosts, reshaping our understanding of host–pathogen interactions. Molecular tools, including PCR-based and genomic approaches, have improved species-level identification, although their availability remains largely restricted to reference centers. Therapeutically, extended-spectrum triazoles and terbinafine have shown the most consistent activity, while adjunctive immunomodulatory strategies, such as imiquimod, are emerging as promising alternatives.

However, these advances sharply contrast with real-world clinical practice. In endemic settings, diagnosis continues to rely on low-cost conventional methods, which, given their sensitivity, should be more widely incorporated into public health screening and active case-finding strategies. Antifungal susceptibility testing lacks clear clinical correlation, and treatment decisions remain largely based on observational studies, in the absence of randomized controlled trials.

**Conclusions and significance:**

CBM exemplifies a neglected tropical disease in which substantial progress in basic science has not translated into improved patient outcomes. Strengthening public health strategies, expanding access to simple and effective diagnostic tools, and prioritizing implementation-focused research are urgently needed to bridge this persistent gap.

## Author summary

Chromoblastomycosis (CBM) is a chronic fungal infection that mainly affects people living in rural and low-resource settings in tropical regions. Although it has been known for over a century, cases are increasingly reported in new geographic areas, including high-income countries, suggesting that its true distribution is underestimated. Despite its impact on patients’ quality of life, the overall burden of disease remains poorly quantified.

In this review, we highlight recent advances in the understanding of CBM. Molecular tools have expanded the identification of causative fungal species and improved knowledge of their biology. New diagnostic techniques and treatment options, including newer antifungal drugs and immunomodulatory therapies, are emerging. However, these advances are largely confined to specialized centers and have limited impact in endemic regions.

In daily practice, diagnosis still relies on simple, low-cost methods, and treatment decisions are often based on clinical experience rather than robust clinical trials. As a result, many patients continue to face delayed diagnosis and suboptimal care. CBM illustrates a major gap between scientific progress and real-world application. Improving outcomes will require stronger public health strategies, wider access to simple diagnostic tools, and research focused on practical solutions that can benefit affected populations.

## Introduction

Chromoblastomycosis is an implantation fungal infection and neglected tropical disease caused by dematiaceous fungi that live in soil and on wood and plants [1]. The disease can impact immunocomponent and immunocompromised persons, most commonly in tropical and subtropical regions. The most common causes of chromoblastomycosis are *Fonsecaea pedrosoi*, *Fonsecaea* monophora, *Fonsecaea* nubica, *Cladophialophora carrionii*, *Rhinocladiella aquaspersa*, *Exophilia* species, and *Phialophora verrucosa*. However, species distribution varies by region and climatic conditions. Diagnosis is often delayed years before treatment is started, leading to reluctant disease that can result in long-term complications including squamous cell carcinoma [1].

Despite growing scientific interest in chromoblastomycosis over the past two decades, most advances have been concentrated in highly specialized areas, particularly in molecular research [1,2]. These include whole-genome sequencing, the development of PCR-based tools for species-level identification, and the expansion of molecular epidemiology studies across endemic regions such as Brazil, China, and Madagascar, providing new insights into pathogen diversity, virulence, and environmental reservoirs [3–8]. While these contributions have significantly improved our understanding of the biology and ecology of the causative agents, they have had limited impact on the realities of patient care in endemic settings [1,3,7,9].

A substantial gap persists between scientific progress and its translation into public health strategies. Accessible point-of-care diagnostics remain scarce, therapeutic decisions are still largely unsupported by randomized clinical trials, and many endemic countries lack structured programs or consistent access to antifungal treatment. Bridging this divide requires a shift toward clinically oriented and implementation-focused research [9–12]. In this context, the present review aims to critically examine the major advances in chromoblastomycosis over the past 20 years, while highlighting the unmet needs that continue to hinder effective diagnosis, treatment, and control of this neglected disease.

## Epidemiology

### What is the current global burden of chromoblastomycosis, and what has changed in epidemiologic understanding in the last 10 years?

The burden and distribution of chromoblastomycosis is not well-understood globally due to substantial gaps in country-level surveillance. In the World Health Organization’s (WHO) global 2025 report on neglected tropical diseases, only one country (Madagascar) reported cases of chromoblastomycosis through an established surveillance system. At least 11 countries reported unknown endemicity status of chromoblastomycosis in the report [13].

The most comprehensive understanding of the burden of chromoblastomycosis comes from a systematic literature review, identifying 7,700 cases. Most cases (34%) occurred in South America and were men with a median age of 53. *Fonsecaea* species were the most common causative fungus [3].

Chromoblastomycosis occurs most commonly in tropical and subtropical areas, but disease can occur anywhere where traumatic injury leads to inoculation of the fungus [1,3]. Interestingly, among Native Americans and Alaska Natives in the United States during 2016–2022, the Alaska region had the second highest prevalence rate (14 per 1,000,000 patients) of chromoblastomycosis and phaeohyphomycotic abscesses, potentially from higher outdoor exposure and trauma [14].

The approach to integrating chromoblastomycosis into other NTD, skin, and other disease programmatic work has progressed, which can lead to better epidemiological understanding [15]. In 2025, clinicians from rural Indonesia implemented an approach of leveraging malaria microscopy infrastructure and cross-training front-line healthcare workers on chromoblastomycosis diagnosis [16]. This led to the first cases of chromoblastomycosis ever recorded on a remote Indonesian island [17].

In this way, telemedicine (encompassing remote evaluation by specialists, including dermatologists and infectious disease physicians) has emerged as a critical tool for identifying previously unrecognized cases, particularly in remote and underserved regions. By enabling multidisciplinary clinical assessment, these approaches help overcome geographic and resource-related barriers to diagnosis. Collectively, these observations indicate that chromoblastomycosis remains substantially underdiagnosed worldwide, especially in areas with limited access to specialized expertise, and support the integration of telemedicine platforms as scalable and adaptable strategies to improve early case detection, expand global surveillance, and strengthen diagnostic capacity across diverse and resource-constrained settings [17,18].

### Have new countries or regions reported cases or publications expanding our understanding of endemicity?

Identification of increases or new areas of endemicity of chromoblastomycosis remains relatively unknown with low awareness of the disease and gaps in routine surveillance. However, recent findings suggest that our understanding of chromoblastomycosis is improving. Twenty-three patients with chromoblastomycosis were described from French Guiana and found those at highest risk were coastal-dwelling individuals engaged in agriculture. In Pakistan, where only two cases had previously been reported, a single-center study identified 16 cases during 2016–2022 [19]. A case series of 13 patients with chromoblastomycosis at a single center in Peru more than doubling the cases previously reported country-wide [20]. Fourteen patients in a case series from Taiwan were older than global estimates, and 13 cultures grew *Fonsecaea monophora,* providing further support that this species is the most common cause of chromoblastomycosis in the region [21]*.* In 2025, the first case of chromoblastomycosis caused by *Phialophora europaea* was reported outside of Europe in Japan [22]. The first locally acquired case of chromoblastomycosis in over 30 years in Chile was reported in 2022, and the first documented case in Lebanon occurred in 2024 [23,24]. In 2025, *Fonsecaea nubica* was identified in a patient with locally acquired chromoblastomycosis in India, the first in the country [25]. Case reports from Qatar and Turkey have also been described [26,27].

### What is known about the disability-adjusted life years (DALYs) and the overall burden of disease?

The true burden of chromoblastomycosis, beyond the number of cases, is unknown. Estimates of measures such as disability-adjusted life years (DALYs), a standardized measure in which global health bodies assess the burden of diseases, have not been calculated. The need to determine these values has been highlighted by the 2025 WHO NTD report and by the global chromoblastomycosis working group, specifically through multicenter efforts to characterize disease burden [3,14,28].

### Expanding concepts in the biodiversity of chromoblastomycosis agents

The spectrum of agents causing chromoblastomycosis appears broader and more dynamic than previously recognized. A substantial expansion in documented species diversity has accompanied the adoption of molecular biology techniques, enabling more precise differentiation of closely related fungi [2,3,6,7,29]. In parallel, an increasing number of atypical cases, particularly among immunocompromised patients, have revealed unusual clinical presentations and the involvement of less commonly reported or emerging species [3,29]. Several unexpected agents have also been implicated, including *Aureobasidium pullulans*, *Rhytidhysteron* spp., *Chaetomium funicola*, *Catenulostroma chromoblastomycosum*, *Kirschsteiniothelia* spp., and *Alternaria* spp., further underscoring the expanding etiologic landscape. This increase in reports poses diagnostic challenges, as cases must be clinically compatible and supported by the demonstration of typical muriform bodies, beyond fungal identification alone, particularly to avoid misclassification as phaeohyphomycosis [30–35]. Nevertheless, despite this growing heterogeneity, *Fonsecaea* species remain by far the most important and consistently documented etiologic agents of chromoblastomycosis worldwide (*F. pedrosoi, F. monophora, F. nubica,* and *F. pugnacius*), followed by *Cladophialophora carrionii*, while species within the genera *Phialophora* and *Rhinocladiella* account for a smaller but well-recognized proportion of cases across diverse geographic settings. Importantly, these taxa remain the fungal agents most strongly associated with chromoblastomycosis, with their role supported by extensive clinical, epidemiological, and molecular evidence [2,3,6,7,29]. More recently, additional agents such as *Cyphellophora ludoviensis* and *Rhinocladiella tropicalis* have been described [2]. Importantly, molecular epidemiology has also redefined the classification of previously reported agents [36,37]. For instance, in Madagascar, a proportion of cases historically attributed to *Fonsecaea pedrosoi* are now likely to represent *Fonsecaea nubica* [38]. Similarly, in the Indian state of Kerala, molecular analysis of nine cultured isolates demonstrated that all *Fonsecaea* strains were *F. nubica* based on CDC42 phylogeny, alongside non-*Fonsecaea* isolates including *Medicopsis romeroi*, *Cladosporium* spp., and *Lasiodiplodia* spp., highlighting the increasing taxonomic refinement and regional variability unveiled through modern approaches [39].

A global consortium can conduct prospective, multicenter studies focused on the molecular identification of chromoblastomycosis agents across all continents, thereby generating standardized, high-resolution epidemiological data and advancing the understanding of the global distribution and diversity of causative species. Fig 1 describes the phylogeny of representative species within the order Chaetothyriales, highlighting the biodiversity of chromoblastomycosis agents.

**Fig 1:**
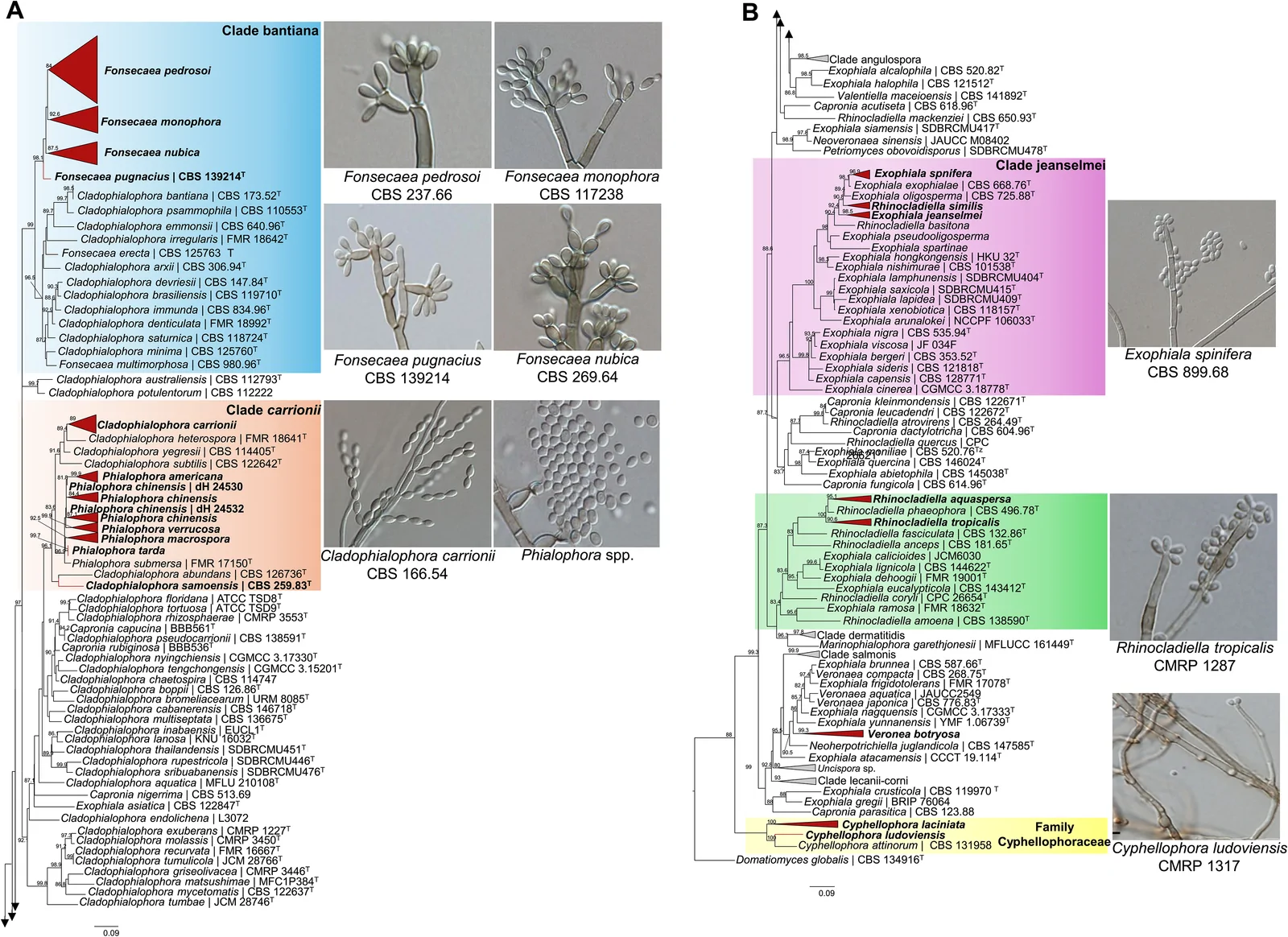
Biodiversity of CBM agents. Phylogeny of a representative selection of species of the Chaetothyriales, based on the confidently aligned LSU and ITS sequences constructed by the maximum likelihood method implemented in Iqtree v2.4.0. Bootstrap values were calculated from 10,000 replicates; values > 80% are shown. Morphologies of the species concerned are shown at the right. Domatiomyces globosa CBS 134916T was used as the outgroup. Names in bold indicate Chromoblastomycosis agents identified based on molecular data from the literature. The picture of *Phialophora* spp. belongs to the strain *P. macrospora* CBS 237.37. The pictures were extracted from the Atlas of Clinical Fungi (https://www.atlasclinicalfungi.org/. **A.** Heportrichiellaceae family: Bantina and Carrionii clades; **B.** Heportrichiellaceae family: Jeanselmei clade and Cyphellophoraceae family.

## Clinical aspects

### Beyond Carrion: Do we need a new clinical classification for CBM?

Chromoblastomycosis arises after traumatic implantation of fungal elements into exposed skin, evolving from an initial macule to papule and progressively to nodular, verrucous, plaque, or tumoral lesions, often with polymorphic features [1,3,6,7,29]. Early stages are frequently overlooked, allowing slow progression over months to decades, with different lesion types commonly coexisting without necessarily indicating greater severity [1,3,6,7,29,40]. Lesions predominantly affect the lower limbs and hands, typically remaining localized but capable of spreading by contiguity, autoinoculation, or via lymphatic pathways in a sporotrichoid pattern [1,3,6,7,21,29,40].

Clinically, the disease is best categorized using the modified Carrion classification, which organizes lesions into nodular, tumoral, verrucous, plaque, and cicatricial forms, facilitating standardized description [41]. Large-scale data from a review of 7,700 cases further confirm the predominance and coexistence of these patterns, reinforcing chromoblastomycosis as a chronic, heterogeneous, and slowly evolving disease [3]. Fig 2 illustrates the principal dermatological manifestations of chromoblastomycosis as originally described by Carrion. Figs 2 and 3 illustrate the spectrum of dermatological patterns of chromoblastomycosis according to the Carrion classification, including atypical clinical presentations.

**Fig 2:**
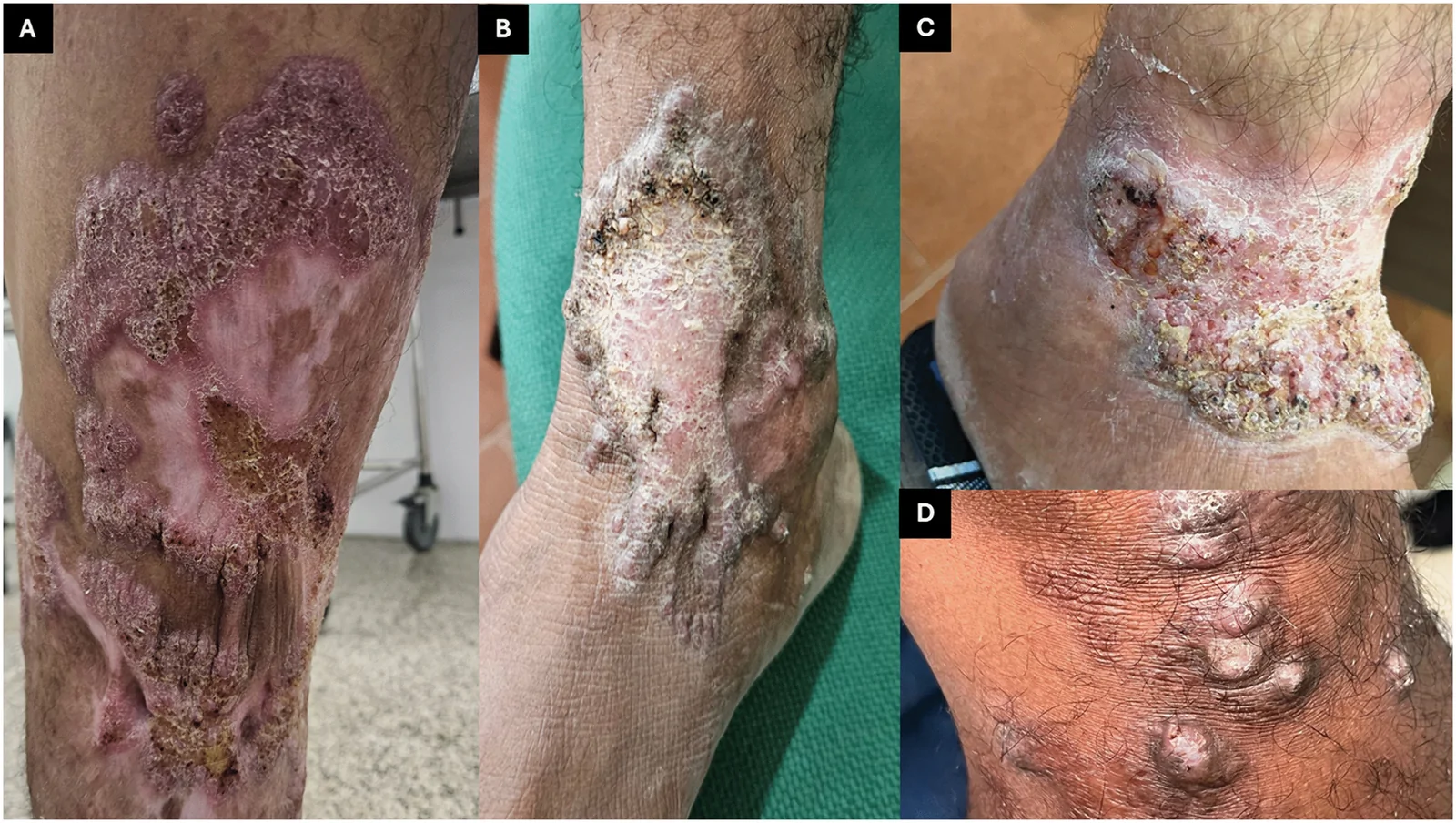
Clinical patterns of CBM lesions. **(A–C)** slightly elevated infiltrated lesion with irregular contours, reddish to violaceous coloration, and a scaly surface; often presenting black dots that correspond to transepidermal elimination sites rich in muriform bodies. **(D)** Isolated subcutaneous nodules.

**Fig 3:**
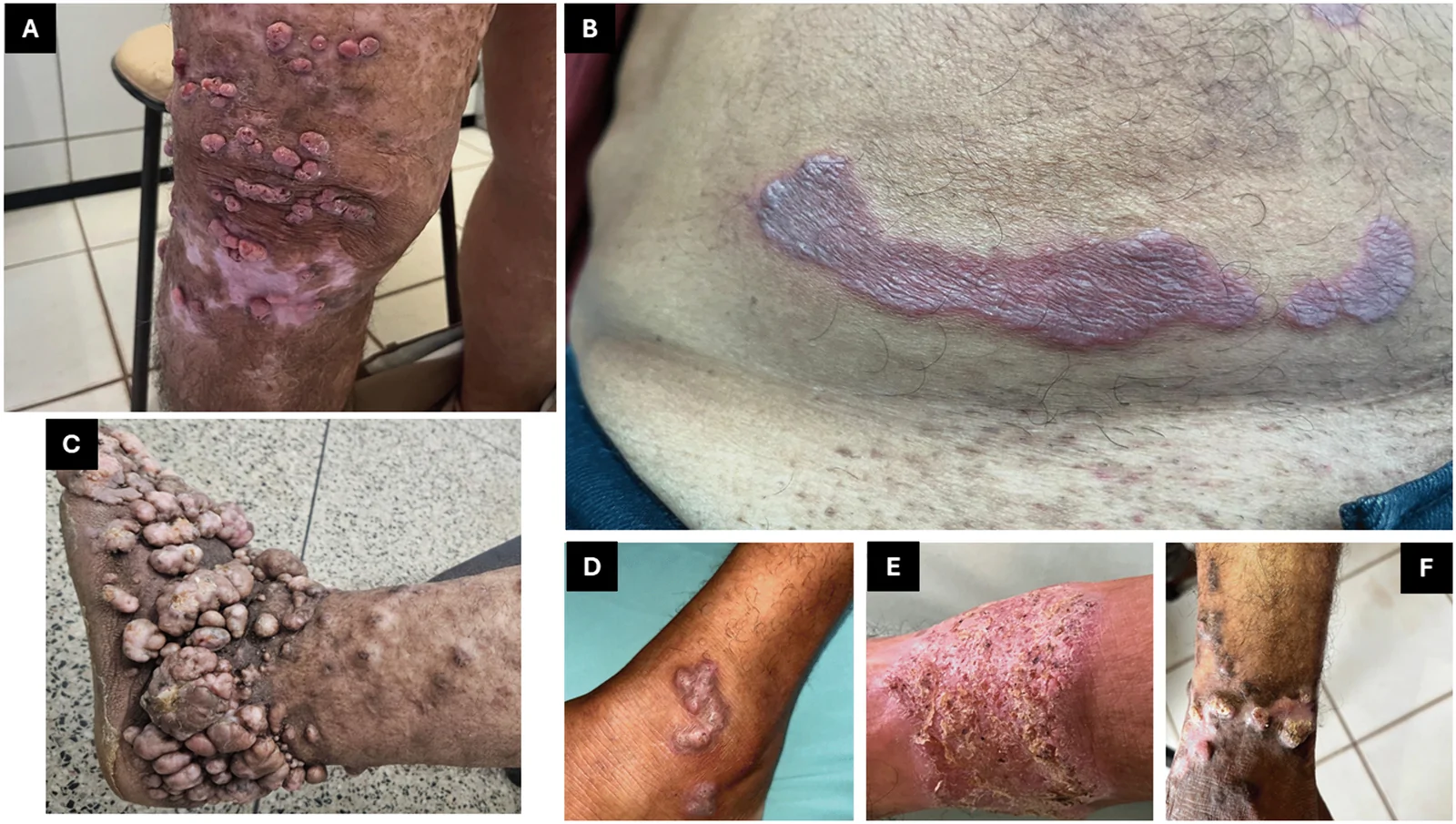
Broad morphological spectrum of CBM. Nodular lesions may appear moderately elevated, soft, and pink to violaceous in color **(A)**, while verrucous plaques can show an atypical psoriasiform or edematous pattern **(B)**. More advanced forms include prominent nodular or tumor-like lesions with a papillomatous surface, often lobulated and resembling a “cauliflower” appearance **(C)**. Disease progression may involve confluent nodules evolving into verrucous plaques **(D)**. Additional presentations include markedly hyperkeratotic, scaly verrucous plaques with black dots, corresponding to transepidermal elimination of muriform bodies **(E)**, as well as scaly verrucous nodules **(F)**.

An increasing number of reports have highlighted the emergence of atypical dermatological patterns in chromoblastomycosis, suggesting a broader clinical spectrum than traditionally recognized. These include discrete or confluent papular lesions with intervening hypopigmented skin and occasional pustules, as well as less conventional presentations such as pseudovacuolar patterns, eczematous forms, phagedenic ulcers, and lesions mimicking palmoplantar psoriasis [42–45]. Collectively, these variants reinforce the polymorphic nature of the disease and underscore the need for heightened clinical suspicion, particularly in endemic areas where non-classical presentations may contribute to delayed diagnosis, suggesting that the traditional Carrion classification may no longer fully capture the expanding clinical spectrum of chromoblastomycosis and that a revised framework should be considered [42,45].

### Beyond the classical host: Is chromoblastomycosis no longer restricted to the immunocompetent?

Although the majority of chromoblastomycosis cases are reported in immunocompetent individuals, an increasing number of cases have been described in patients with varying degrees of immunosuppression [3,29]. These include individuals undergoing chemotherapy for solid or hematologic malignancies, patients with autoimmune diseases such as systemic lupus erythematosus and rheumatoid arthritis, people living with HIV, and even those with conditions such as celiac disease [46–49]. Notably, a growing body of literature has documented CBM in solid organ transplant recipients, highlighting a shift in the epidemiological profile of the disease and suggesting that host immune status may play a more significant role than previously appreciated [50,51].

### Chromoblastomycosis and co-endemic infections: Coincidence or an underrecognized pattern?

Given that chromoblastomycosis is a neglected tropical disease occurring in regions where multiple infectious diseases coexist, the overlap with other endemic conditions is likely underrecognized [3,29]. Concomitant subcutaneous infections, including leprosy, mycetoma, cutaneous histoplasmosis, cutaneous leishmaniasis, actinomycosis, and filariasis may occur alongside CBM, as well as systemic infections such as visceral leishmaniasis, paracoccidioidomycosis, and syphilis [52–61]. This convergence of diseases is probably more frequent than currently reported, reflecting not only gaps in the literature but also significant deficiencies in healthcare access and diagnostic capacity. Such overlap should be interpreted as a marker of systemic neglect, particularly in populations living in conditions of extreme poverty [62,63]. Clinicians should systematically screen for other poverty-associated endemic skin diseases through careful clinical evaluation, particularly when facing atypical presentations.

### Neglect, poverty, and delay: The perfect storm of complications in CBM

CBM is a chronic and often progressive infection, in which delayed diagnosis or suboptimal treatment may lead to significant morbidity and irreversible sequelae. Secondary bacterial infection is frequent in long-standing lesions, contributing to increased pain, exudation, malodor, and additional inflammatory burden, thereby complicating both local wound care and systemic management [3,53,64,65]. In advanced stages of chromoblastomycosis, chronic lymphedema may develop and, in severe cases, progress to joint stiffness and ankylosis as a result of prolonged inflammation and progressive tissue damage [1,3,7,53,65,66]. Persistent lymphatic compromise, particularly in lower-limb disease, can lead to extensive edema and, ultimately, elephantiasis, marked by disfiguring enlargement, dermal fibrosis, and significant functional limitation [67].

Malignant transformation is a rare but highly severe complication of chromoblastomycosis. Azevedo and colleagues described 7 cases in Brazil and reviewed 10 additional cases, with a mean disease duration of 23 years (range 5–36 years) before carcinogenesis, which occurred regardless of prior antifungal therapy and frequently required limb amputation [68]. In a larger dataset, Santos and colleagues (2021) identified 22 malignant cases among 7,700 reported CBM patients. Although uncommon, this outcome represents a devastating consequence of long-standing disease [1,3,66,68–70].

## Diagnosis

### Do we really need high-tech tools to diagnose chromoblastomycosis in resource-limited settings?

The diagnosis of CBM is fundamentally straightforward and relies primarily on the identification of muriform cells through direct mycological examination or histopathology. Large clinical-epidemiological studies from reference centers have demonstrated variable utility of histopathology, whereas direct examination with 10% potassium hydroxide (KOH) remains a simple, low-cost, and highly sensitive method, with detection rates approaching 95% [1,3]. Skin scrapings containing crusts, cellular debris, and tissue fragments are easily processed using KOH (10–40%), allowing visualization of characteristic muriform cells, round to polyhedral, brownish structures measuring 5–12 μm, often arranged singly or in clusters [1,3,7,40]. Dermoscopy, a non-invasive tool, can reveal the characteristic black dots associated with chromoblastomycosis and provide clues to inform the highest-yield locations on the skin to collect skin scrapings or a biopsy. Culture on routine media such as Sabouraud dextrose agar further supports diagnosis, with most etiologic agents producing slow-growing, pigmented colonies that evolve from dark green to velvety, progressively melanized forms over incubation periods that may extend up to six weeks [1,3,6,7,40,65,66].

Histopathological examination plays an important role in settings where fungal isolation is not routinely available. Although tissue responses are non-specific and may resemble those seen in other implantation mycoses, the presence of characteristic muriform cells is diagnostic [1,3,6,7,40]. These structures may be observed within multinucleated giant cells on routine hematoxylin–eosin staining, while special stains such as Gomori-Grocott and Fontana-Masson increase sensitivity in cases with scarce fungal elements. Additional histopathological features commonly include tuberculoid granulomas, suppurative granulomas, hyperkeratosis, pseudoepitheliomatous hyperplasia, pyogranulomatous inflammation, and irregular acanthosis alternating with areas of atrophy [1,3,6,7,9,40,65,66]. Fig 4 shows the main histological findings of CBM.

**Fig 4:**
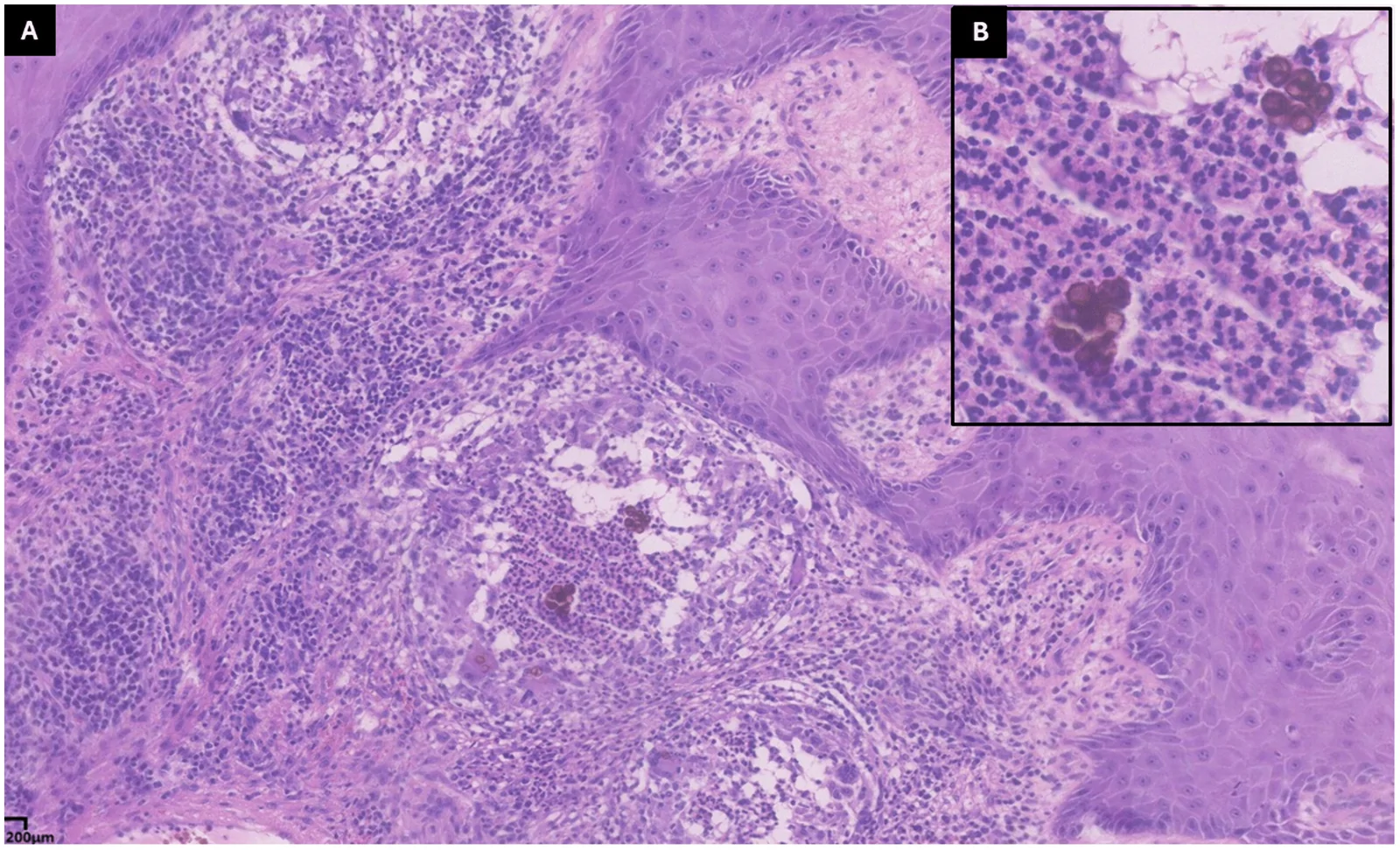
The main histological findings of CBM. **(A)**: Histopathology (H&E, 100×). The epidermis shows marked acanthosis with irregular elongation of rete ridges. In the dermis, there is a dense and diffuse lymphomononuclear inflammatory infiltrate composed of lymphocytes and histiocytes, the latter frequently arranged in well-formed granulomas. These granulomatous structures are accompanied by scattered neutrophils, reflecting a suppurative component. Multinucleated giant cells are present within the granulomas. **(B)**. H&E (400×): Brownish, oval fungal elements arranged often clustered and occasionally arranged in chains, “copper penny” (stacked coin) configuration, consistent with muriform (sclerotic) bodies characteristic of chromoblastomycosis.

Molecular diagnostic methods in chromoblastomycosis remain largely confined to research and reference laboratories, with limited impact on routine clinical practice, where diagnosis can be readily achieved with high sensitivity through direct microscopic examination of lesion scrapings. In this setting, molecular tools are primarily used for species-level identification, typically from pure culture isolates, and have been essential for refining taxonomy and uncovering cryptic diversity within the *Fonsecaea* complex [2,71–74].

To improve accessibility and reduce turnaround times, PCR-based techniques, such as PCR-RFLP and multiplex assays targeting ITS and LSU regions, have been developed, allowing more rapid and practical species identification while reducing reliance on sequencing [73,74]. More recently, simplified molecular platforms, including loop-mediated isothermal amplification (LAMP) and rolling circle amplification (RCA), have been proposed as potential point-of-care approaches, aiming to bridge the gap between high-complexity diagnostics and the needs of endemic, resource-limited settings [75–78].

## Beyond the MIC: Is antifungal susceptibility testing clinically relevant in CBM?

Despite the absence of standardized interpretive breakpoints for most agents causing chromoblastomycosis, clinical evidence consistently supports itraconazole, voriconazole, posaconazole, and terbinafine as the most active agents, based on cumulative case series [1,6,40,53,66]. Early studies using macro- and microdilution methods demonstrated low minimum inhibitory concentrations (MICs) for itraconazole and terbinafine against the main CBM pathogens [79,80]. Subsequently, Najafzadeh and colleagues (2010) reported in vitro susceptibility data for *Fonsecaea pedrosoi*, *F. monophora*, and *F. nubica*, showing a descending order of antifungal activity as follows: posaconazole (0.063 µg/mL), itraconazole (0.125 µg/mL), isavuconazole (0.25 µg/mL), and voriconazole (0.5 µg/mL), highlighting the potential added clinical value of newer-generation azoles [81].

Similar in vitro susceptibility patterns have been observed for *Cladophialophora carrionii* and its related species *C. yegresii*, as well as *Phialophora verrucosa* and less common agents such as *Cyphellophora* spp., all demonstrating favorable activity with posaconazole, itraconazole, terbinafine, and voriconazole [82–85]. In contrast, amphotericin B and echinocandins generally show limited in vitro efficacy and should not be considered optimal therapeutic options for CBM [81–85].

More recently, Smith and colleagues (2025) established epidemiological cutoff value (ECVs) for key antifungals against *Fonsecaea pedrosoi*, including itraconazole, voriconazole, and posaconazole (0.5 µg/mL), isavuconazole (1 µg/mL), terbinafine (0.25 µg/mL), and amphotericin B (8 µg/mL), aligning with earlier susceptibility data. ECVs for *Fonsecaea monophora* which were established for itraconazole (2 μg/mL), voriconazole (0.25 μg/mL), posaconazole (1 μg/mL), isavuconazole (0.25 μg/mL), and terbinafine (0.5 μg/mL) allow exploration of differences in susceptibility patterns within the *Fonsecaea* genus. For example, the *F. monophora* ECV for itraconazole is 2-fold dilutions higher than against *F. pedrosi* (2 μg/mL versus 0.5 μg/mL). These thresholds, derived from a multicenter international initiative, provide an important reference framework for characterizing susceptibility patterns and tracking emerging resistance [86]. Importantly, they offer clinicians and researchers a practical tool to identify isolates with reduced susceptibility, inform therapeutic reassessment, and support the investigation of suspected clinical resistance in cases of treatment failure [81,86].

A major limitation of antifungal susceptibility testing (AST) in chromoblastomycosis lies in its ability to accurately reproduce the pathogen’s biological state within the host [1,87]. Ideally, testing should be performed using muriform (sclerotic) cells rather than the filamentous phase, as this form better reflects in vivo conditions. Addressing this gap, Takahashi and colleagues (2026) evaluated 43 clinical isolates of *Fonsecaea* spp. using both conidial and sclerotic forms, demonstrating that posaconazole exhibited the greatest antifungal activity, with low geometric mean MICs in both morphotypes. Importantly, clinical correlation revealed that higher itraconazole MIC values were associated with poorer patient outcomes, whereas lower MICs were observed in cases with favorable responses [88]. These findings reinforce the relevance of morphotype-specific testing and support the potential role of posaconazole and voriconazole as alternative options, particularly in patients with suboptimal response to itraconazole [86–88].

Moreover, there is a clear need for EUCAST and CLSI committees to advance the standardization of antifungal susceptibility testing methodologies, providing clinicians with more reliable guidance for therapeutic decision-making, even in settings where only provisional epidemiological cutoff values are currently established [86–88].

## Treatment

### Disruptive advances in physical, antifungal, and immunomodulatory therapy

#### Physical therapy.

The management of chromoblastomycosis is complex and typically requires a multimodal approach combining both physical and pharmacological strategies [1,6,7,29]. Antifungal therapy remains the cornerstone of treatment, but physical interventions, such as surgical excision, cryotherapy, or thermotherapy, may be employed either alone in early-stage disease or in combination with antifungal agents in more advanced cases, depending on lesion extent and severity [6,89–91]. While small, localized lesions can often be effectively managed with surgical removal, outcomes are less well characterized in terms of long-term follow-up. In contrast, moderate to severe disease is frequently marked by chronic, refractory lesions, in which achieving complete clinical resolution remains challenging despite prolonged and combined therapeutic regimens [92,93].

Among contemporary physical treatment modalities, photodynamic therapy (PDT) has emerged as a promising adjunctive approach in the management of chromoblastomycosis (CBM) [1]. Experimental studies have demonstrated significant in vitro efficacy, including marked growth inhibition of *Fonsecaea pedrosoi* and *Cladophialophora carrionii* using methylene blue, as well as encouraging results with novel agents such as the porous organic cage photosensitizer (COP1T-HA) against *Fonsecaea monophora* and new methylene blue against multidrug-resistant *Fonsecaea nubica* [94–96]. *In vivo* models, including a *Balb/c* mouse paw infection model with *F. monophora*, further suggest that potassium iodide may enhance the antifungal effect of methylene blue-mediated PDT. These experimental findings are supported by clinical observations, in which PDT has demonstrated meaningful therapeutic benefit, particularly in severe or refractory cases [97]. In small clinical series, patients treated with topical methylene blue-based PDT showed substantial lesion reduction, with significant clinical and mycological improvement after repeated applications. Overall, PDT appears to be a safe, well-tolerated, and potentially effective strategy, especially when integrated into combined treatment regimens for difficult-to-treat CBM [98–103].

#### Antifungal therapy.

A critical limitation in the management of chromoblastomycosis (CBM) is the absence of randomized, controlled comparative clinical trials, a persistent gap that reflects the broader challenges faced by neglected tropical diseases. Over the past four decades, the wide range of chemical therapies employed underscores the lack of standardized, evidence-based treatment approaches [1]. Historically, numerous agents including arsenic, mercury, gold, antimonials, bismuth, methylene blue, sulfonamides, and others were used with limited or inconsistent success [1,52,104]. The introduction of amphotericin B in the 1950s and 5-fluorocytosine in the late 1960s represented incremental advances, but it was only with the development of azoles in the 1980s that more consistent therapeutic responses began to emerge [105–107]. Since its introduction, itraconazole has become the cornerstone of pharmacological therapy for chromoblastomycosis, typically administered at doses ranging from 200 to 400 mg/day. However, therapeutic outcomes remain highly heterogeneous and are influenced by multiple factors, including socioeconomic conditions, disease severity, lesion morphology, etiologic species, dosing strategies, and treatment duration [1,7,40,57,60,108,109]. Clinical series from reference centers illustrate this variability: some cohorts have reported complete clinical and mycological cure in a minority of patients (22%–80%), with a larger proportion achieving only partial improvement despite combined approaches, while others have documented modest cure rates after several months of therapy [7,29,53,57,60,65,66,109]. Additionally, long-term follow-up studies highlight challenges such as treatment discontinuation and loss to follow-up, often accompanied by suboptimal response rates with standard dosing regimens. Successful therapy with a combination of itraconazole and flucytosine has been reported, particularly in severe, long-standing disease, but data are limited and logistics and costs with flucytosine may prevent its use. Collectively, these findings reinforce that, although itraconazole remains the drug of choice, its effectiveness is inconsistent, underscoring the critical need for optimized strategies and stronger evidence to guide treatment [7,29,53,57,60,65,66,109–111].

Terbinafine is generally regarded as a second-line option, largely due to the more limited clinical experience with its use. Early studies from Madagascar demonstrated its efficacy in CBM, particularly in recent lesions and infections caused by *Cladophialophora* spp., with prolonged therapy (500 mg/day for 6–12 months) resulting in rapid clinical improvement and cure in a substantial proportion of patients, including refractory cases [112,113]. However, subsequent smaller reports have shown heterogeneous outcomes, and although some groups employ terbinafine as an alternative therapeutic strategy, its use remains less consistent compared to first-line azole-based regimens [29,114–118]. Terbinafine combined with itraconazole has been used successfully to treat recalcitrant disease in very few case reports, but larger studies are unavailable to truly understand its role in treating chromoblastomycosis [116,118]. Yap and colleagues reported 11 patients treated with terbinafine plus itraconazole, with adjunctive cryotherapy or electrocautery in some cases. Clinical cure was achieved in 54.5% of patients and partial response in 18.2%. Three patients discontinued treatment after two months [116].

Advanced-generation azoles, such as voriconazole and posaconazole, exhibit favorable in vitro activity against the main causative agents of chromoblastomycosis, along with more predictable pharmacokinetic and pharmacodynamic profiles compared with itraconazole. However, clinical experience remains limited. For voriconazole, approximately ten cases have been reported, most involving patients with prior treatment failure to itraconazole, terbinafine, or physical modalities, thus reflecting a predominantly refractory population. Treatment duration was generally less than one year, with outcomes showing clinical cure in a subset of cases, partial improvement in others, and occasional relapse despite initial response [33,99,119–124].

Similarly, data on posaconazole are restricted to a small number of published cases, but available reports suggest encouraging results, with most patients achieving clinical cure after treatment courses of up to 12 months. Its lipophilic profile and enhanced penetration into soft tissues further support its potential role in CBM management [125–128]. In contrast, there are currently no clinical reports evaluating the use of isavuconazole in refractory chromoblastomycosis, highlighting an additional gap in the therapeutic landscape. Table 1 describes published cases of CBM treated with voriconazole and posaconazole.

**Table 1:** Clinical description and outcome of published CBM cases treated with voriconazole and posaconazole.

[Ref] Year published/ No patients	Age	Time of illness	Clinical aspects	Agent	Previous treatment	VOR/ POS dosis	Treatment duration (VOR/POS)	Combined therapy	Outcome
**VOR**									
[119] 2011/ 1	55 y/ male	35 y	Large, reddish, cicatricial plaque on the left lower aspect of his leg, and several tumorous lesions surrounded the plaque	*F. monophora*	ITZ	400 mg/d	18 mo	No	Cure
[120] 2011/ 3	77 y/ male	21 y	Polymorphic skin lesions, including nodular, tumoral, verrucous, cicatricial, and plaque-type lesions on his right lower limb and a small area of the dorsum	*F. pedrosoi*	ITZ, TERB, POS	200 mg/d	11 mo	No	Clinical cure and relapse
	59 y/ male	10 y	Verrucous plaques on the entire left lower limb	*F. pedrosoi*	ITZ, TERB, cryotherapy	400 mg/d	12 mo	No	Clinical cure and relapse
	44 y/ male	20 y	Extensive verrucous plaques disseminated on the face, upper right limb, large areas of the trunk and lower left limb	*F. pedrosoi*	ITZ, AMB	400 mg/d	12 mo	No	Cure
[99] 2012/ 1	55 y/ male	13 y	Erythematous plaque surrounded by verrucous hyperplasia on the medial side of his right arm	*F. monophora*	ITZ, TERB	200 mg/d	2 mo	VOR, TERB, PDT	Improvement
[121] 2015/ 1	53 y/ male	20 y	Multiple keratotic nodules coalescing into verrucous plaques on the right buttock (he had rheumatoid arthritis)	*F. pedrosoi*	ITZ, TERB	400 mg/d	5 mo	Local hyperthermia	Cure
[122] 2016/ 1	48 y/ male	5 y	Nodular lesion on the lower left limb and lumbar region, evolving to progressive increase in erythematous verrucous plaques with areas of atrophy	*F. pedrosoi*	ITZ, TERB, thermotherapy; cryosurgery	400 mg/d	4 w	No	Improvement
[123] 2018/ 1	52 y/ male	30 y	Plaques, nodules, ulcers on left leg	*F. pedrosoi*	systemic antifungals, PDT and cryotherapy	400 mg/d	NA	No	NA
[33] 2024/ 1	46 y/ female	1 y	Dark, verrucous, and ulcerative lesions on the legs (she is a kidney transplanted patient)	*Alternaria infectoria*	ITZ, cryotherapy	200 mg/d	5 mo	No	Cure
[124] 2025/ 1	69 y/ female	5 mo	Papules on the dorsum of her left hand; nodules on the left lower limb accompanied by ulceration	*F. monophora*; co-infection *Scedosporium dehoogii*	no	400 mg/d	4 w	No	Improvement
**POS**									
[125] 2005/ 5	45 y/ female	12 y	NA	*F. pedrosoi*	ITZ, TERB	800 mg/d	12 mo	No	Cure
	69 y/ male	4 y	NA	*F. pedrosoi*	ITZ, TERB	800 mg/d	12 mo	No	Improvement
	55 y/ male	3 y	NA	*F. pedrosoi*	ITZ, TERB	800 mg/d	12 mo	No	Cure
	47 y/ male	16 y	NA	*F. pedrosoi*	ITZ, TERB, 5FC, SAP	800 mg/d	12 mo	No	Improvement
	69 y/ male	29 y	NA	*F. pedrosoi*	ITZ, TERB, 5FC	800 mg/d	12 mo	No	Cure
	53 y/ male	8 y	NA	*F. pedrosoi*	ITZ, TERB	800 mg/d	12 mo	No	Cure
[126] 2010/ 1	58 y/ male	3 mo	4 nodules on the right elbow, 3 of them crusting. (he had a kidney and pancreas transplant)	*C. carrionii*		800 mg/d	4.5 mo	No	Cure
[127] 2023/ 1	61 y/ NA	37 y	Verrucous plaque on dorsum of the hand	*Fonsecaea spp.*	ITZ, cryotherapy	300 mg/d	12 mo	IMQ	Cure
[128] 2025/ 1	26 y/ male	2 y	Itchy, erythematous, mildly scaly plaque involving the lower lip, angles of the mouth, and upper part of the chin	*Exophiala spinifera*	ITZ, VOR, TERB	300 mg/d	12 mo	No	Cure

VOR, voriconazole; POS, posaconazole; TERB, terbinafine; PDT, photodynamic therapy; IMQ, topical Imiquimod; NA, not available; y, years; mo: months.

### Can host immune modulation redefine therapeutic outcomes?

The immunological landscape provides a compelling rationale for the exploration of adjunctive immunomodulatory strategies in chromoblastomycosis. Clinical evidence suggests that redirecting the host response toward a more effective Th1-driven profile may enhance fungal clearance and improve therapeutic outcomes, particularly in patients with refractory disease [129]. In this context, Azevedo and colleagues (2008) reported a severe case of CBM unresponsive to itraconazole that showed marked clinical improvement following the addition of weekly intramuscular β-1,3-glucan derived from *Saccharomyces cerevisiae*. This intervention was associated with immune modulation characterized by increased endogenous IFN-γ production and reduced IL-10 levels [130]. Notably, the immunostimulatory properties of β-glucan had previously been demonstrated in experimental models of *Leishmania donovani* infection and in clinical use for paracoccidioidomycosis, further supporting its potential role as an adjunctive immunotherapeutic strategy in difficult-to-treat CBM [131,132].

With the improved characterization of the immune response in chromoblastomycosis, new insights into immunoregulatory strategies have emerged. De Sousa and colleagues (2014) explored the use of imiquimod, a Toll-like receptor 7 (TLR7) agonist that activates both innate and adaptive immunity, reporting the first cases of its use in combination with antifungal therapy. This approach was associated with enhanced local inflammatory responses, promoting pathogen clearance and contributing to clinical resolution, thereby reinforcing the potential role of host-directed therapies in refractory CBM [127,133–135].

Acitretin is a second-generation retinoid with immunomodulatory effects that acts by modulating cytokine pathways, reducing cellular proliferation and inflammation while promoting tissue differentiation [136]. Its use in chromoblastomycosis remains very limited, with only 3 isolated reports describing its combination with topical 5% imiquimod leading to significant lesion improvement, thus characterizing acitretin as a potential rescue therapy [137,138]. Table 2 describes clinical cases of CBM treated with immunomodulators (glucan, imiquimod, acitretin).

**Table 2:** Clinical description and outcome of published CBM cases treated with immunomodulators (glucan, imiquimod, acitretin).

[Ref] Year published/ No patients	Age	Time of illness	Clinical aspects	Agent	Antifungal treatment	Adjuvant therapy	Time of treatment	Outcome
**Glucan**								
[130] 2008/ 1	50 y/ male	27 y	Nodular lesion and progressed to infiltrative plaques on all torsos and face	*F. pedrosoi*	ITZ + TERB	Glucan (beta-1,3 polyglucose) intramuscular	6 mo	Important improvement
**IMQ**								
[133] 2014/ 3	71 y/ male	2 y	Verrucous plaque on the right forearm	*F. pedrosoi*	ITZ 200mg/d	Topical imiquimod 5%	10 mo	Cure
	70 y/ male	3 y	Verrucous plaque on the dorsum of the right hand	*F. pedrosoi*	ITZ 400mg/d + TERB 250mg/d	Topical imiquimod 5%	9 mo	Important improvement
	46 y/ male	6 y	Verrucous plaque on the dorsum of left hand	NA	ITZ 400mg/d + TERB 250mg/d	Topical imiquimod 5%	6 mo	Partial improvement
	71 y/ male	5 y	Verrucous plaque on the right wrist	NA	ITZ 200mg/d + TERB 250mg/d	Topical imiquimod 5%	9 mo	Important improvement
[134] 2020/ 1	45 y/ male	4 y	Erythematous plaque surrounded by verrucous hyperplasia on the dorsum of the left hand	*F. pedrosoi*	Previous ITZ	Topical imiquimod 5% monotherapy	7 mo	Cure
	43 y/ male	9 mo	Verrucous lesion on the back of his right hand	*F. pedrosoi*	No	Topical imiquimod 5% monotherapy	6 mo	Cure
	39 y/ male	2 y	Verrucous lesion on the right wrist	*F. pedrosoi*	Previous ITZ	Topical imiquimod 5% monotherapy	19 mo	Cure
[135] 2021/ 1	72 y/ male	11 mo	Erythema and verrucous plaque on the forearm	*F. monophora*	Previous ITZ	Topical imiquimod 5% monotherapy	3 mo	Cure
[127] 2023/ 1	61 y/	37 y	Verrucous plaque on dorsum of the hand	*Fonsecaea spp.*	ITZ, cryotherapy	Topical imiquimod 5%	12 mo	Cure
**Acitretin**								
[137] 2020/ 2	55 y/ male	35 y	Erythematous, infiltrated, circumscribed, irregular plaques with hyperkeratotic verrucous surface on left leg	*Fonsecaea spp.*	ITZ, FCZ, TERB, KETO, cryotherapy and phototherapy	Topical imiquimod 5% + acitretin 50 mg/d + ITZ	4 mo	Important improvement. Leg was amputated for neoplastic lesion. Cure
	46 y/ male	22 y		*Fonsecaea spp.*	ITZ, TERB, Cryotherapy	Topical imiquimod 5% + acitretin 50 mg/d + ITZ	4 mo	Important improvement
[138] 2023/ 2	65 y/ male	11 y	Verrucous lesions that started on the back and outer side of the right foot	*F. pedrosoi*	ITZ 200mg/d + TERB 250mg/d	Topical imiquimod 5% + acitretin 50 mg/d	5 mo	Important improvement

VOR, voriconazole; POS, posaconazole; TERB, terbinafine; PDT, photodynamic therapy; IMQ, topical Imiquimod; NA, not available; y, years; mo: months.

## Future directions

### What are the major research gaps that need to be addressed in the next decade?

Chromoblastomycosis remains one of the most neglected NTDs with very little research and development funding [12,13,139]. We have described recent updates on the epidemiology, diagnosis, and treatment of this NTD, but further action is needed to prevent disease and long-term complications. Routine surveillance can help monitor for emerging trends and new areas of endemicity and can be combined with other disease surveillance systems. Clinicians and public health officials can include a DALY estimations in routine surveillance to describe the true burden of disease [11,12,15]. Validation of affordable molecular panels (PCR-RFLP/ITS) and standardization of mNGS algorithms for complex or FFPE samples can improve organism identification. Development of further ECVs, and ultimately breakpoints, can help monitor for antifungal resistance and identify appropriate first-line treatments for different causative fungi. Clinicians can conduct prospective studies on voriconazole, posaconazole and isavuconazole, itraconazole plus terbinafine combinations, and pragmatic trials using adjuvant agents such as imiquimod or PDT.

### How can public health strategies and neglected tropical disease policies be strengthened to improve outcomes?

Chromoblastomycosis can be further integrated into other skin NTD and common skin disease surveillance initiatives, and public health strategies to identify disease can center around leveraging microscopy skills in rural or decentralized areas. Training frontline healthcare workers is critical to diagnose infections early to prevent severe morbidity and difficult-to-treat disease. Antifungals commonly used for CBM are unavailable or unaffordable in areas where the disease most commonly occurs [10,140]. National and regional strategies can prioritize sustained, affordable access to antifungals given the long treatment durations required for many patients [11,15,141].

## Conclusions

Despite its formal recognition as a neglected tropical disease by the World Health Organization, CBM remains insufficiently incorporated into national health agendas in many endemic countries. Only a limited number of regions have established dedicated programs that integrate patient care with structured research initiatives, and even these remain largely dependent on local efforts rather than sustained federal support. Bridging this gap will require coordinated public health policies, including the development of low-cost diagnostic laboratories and the training of primary healthcare teams to enable active case finding and early treatment through community-based programs. At the same time, remarkable advances in molecular biology have significantly improved our understanding of CBM pathogens, including their taxonomy, virulence, and environmental distribution. However, these insights have not translated into accessible point-of-care diagnostic tools, underscoring a persistent disconnect between scientific innovation and clinical applicability. This gap continues to hinder effective disease control. Therapeutic strategies remain largely empirical, based on case series rather than robust clinical trials. While itraconazole and terbinafine are widely used, newer triazoles show promise but remain limited by cost and insufficient comparative evidence. Adjunctive immunomodulatory approaches, particularly imiquimod, represent a potential path forward, although rigorous clinical validation is still needed.

**Key learning points**

- Increasing number of reported cases in the last 5 years, including in low-incidence countries. Atypical presentations and cases in immunosuppressed hosts are emerging.
- Advances in genomic taxonomy and PCR-based methods have expanded species identification and improved laboratory characterization of CBM agents.
- Diagnosis remains based on simple, low-cost methods in endemic settings, despite molecular progress. Antifungal susceptibility testing lacks clear bedside relevance and may serve primarily for resistance surveillance.
- CBM remains a neglected tropical disease with persistent gaps between advances in basic science and the implementation of public health strategies for diagnosis and treatment.
- Treatment is largely empirical, based on accumulated clinical experience. Robust, evidence-based therapeutic frameworks are still lacking.

**Key papers**

- Queiroz-Telles F, de Hoog S, Santos DW, Salgado CG, Vicente VA, Bonifaz A, Roilides E, Xi L, Azevedo CM, da Silva MB, Pana ZD, Colombo AL, Walsh TJ. Chromoblastomycosis. Clin Microbiol Rev. 2017 Jan;30(1):233–276. https://doi.org/10.1128/CMR.00032-16.
- Santos DWCL, de Azevedo CMPES, Vicente VA, Queiroz-Telles F, Rodrigues AM, de Hoog GS, Denning DW, Colombo AL. The global burden of chromoblastomycosis. PLoS Negl Trop Dis. 2021 Aug 12;15(8):e0009611. https://doi.org/10.1371/journal.pntd.0009611.
- Smith DJ, Queiroz-Telles F, Rabenja FR, et al. A global chromoblastomycosis strategy and development of the global chromoblastomycosis working group. Bongomin F, ed. *PLoS Negl Trop Dis*. 2024;18(10):e0012562. https://doi.org/10.1371/journal.pntd.0012562.
- Rasamoelina T, Maubon D, Andrianarison M, Ranaivo I, Sendrasoa F, Rakotozandrindrainy N, Rakotomalala FA, Bailly S, Rakotonirina B, Andriantsimahavandy A, Rabenja FR, Andrianarivelo MR, Cornet M, Ramarozatovo LS. Endemic Chromoblastomycosis Caused Predominantly by Fonsecaea nubica, Madagascar^1^. Emerg Infect Dis. 2020 Jun;26(6):1201–1211. https://doi.org/10.3201/eid2606.191498.
- Smith DJ, Melhem MSC, Dirven J, de Azevedo CMPeS, Marques SG, Jacomel Favoreto de Souza Lima B, Vicente VA, Teixeira Sousa MdG, Venturini J, Wiederhold NP, Seyedmousavi A, Dufresne PJ, de Hoog S, Lockhart SR, Hagen F, Santos DWdCL. Establishment of epidemiological cutoff values for *Fonsecaea pedrosoi*, the primary etiologic agent of chromoblastomycosis, and eight antifungal medications. J Clin Microbiol. 2025 May 14;63(5):e0190324. https://doi.org/10.1128/jcm.01903-24.
